# Schwann Cell-Mediated Preservation of Vision in Retinal Degenerative Diseases via the Reduction of Oxidative Stress: A Possible Mechanism

**Published:** 2016

**Authors:** Raziyeh MAHMOUDZADEH, Saeed HEIDARI-KESHEL, Alireza LASHAY

**Affiliations:** 1 Stem Cell Preparation Unit, Eye Research Center, Farabi Eye Hospital, Tehran University of Medical Sciences, Iran

**Keywords:** Schwann Cell, Retinal Degenerative Diseases, Oxidative Stress

## Abstract

After injury to the central nervous system (CNS), regeneration is often inadequate, except in the case of remyelination. This remyelination capacity of the CNS is a good example of a stem/precursor cell-mediated renewal process. Schwann cells have been found to act as remyelinating agents in the peripheral nervous system (PNS), but several studies have highlighted their potential role in remyelination in the CNS too. Schwann cells are able to protect and support retinal cells by secreting growth factors such as brain-derived neurotrophic factor, glial cell line-derived neurotrophic factor, and basic fibroblast growth factor. Retinal degenerative diseases can be highly debilitating, and they are a major concern in countries with an ageing populations. One of the leading causes of permanent loss of vision in the West is a retinal degenerative disease known as age-related macular degeneration (AMD). In the United States, nearly 1.75 million people over the age of 40 have advanced AMD, and it is estimated that this number will increase to approximately 3 million people by 2020. One of the most common pathways involved in the initiation and development of retinal diseases is the oxidative stress pathway. In patients with diabetes, Schwann cells have been shown to be able to secrete large amounts of antioxidant enzymes that protect the PNS from the oxidative stress that results from fluctuations in blood glucose levels. This antioxidant ability may be involved in the mechanism by which Schwann cells are able to promote reconstruction in the CNS, especially in individuals with retinal injuries and degenerative diseases.

## INTRODUCION

After mechanical injury to the central nervous system (CNS) or the progression of degenerative diseases of the CNS, regeneration is often inadequate because the adult CNS is not capable of extensive regeneration. However, one exception is myelin regeneration, which is an example of stem/precursor cell-mediated CNS rehabilitation. This process involves the production of multilayer sheaths of myelin (which are outgrowths of glial cells) around the injured axons, and it can preserve the function of demyelinated neurons and support neurons (1). Research has shown that Schwann cells (a type of glial cell) can produce myelin in the peripheral nervous system (PNS) and, more recently, they have been found to play the same role in CNS (based on experimental animal models and cases of demyelinating disorders of the CNS in humans) (2). PNS Schwann cells are good candidates for autologous grafts to demyelinated CNS axons in patients with CNS injuries (3) because they are not recognized as foreign by the immune system; they are an easily accessible source of cells (4); they can proliferate in cell culture; and they have a good survival rate after transplantation (5). Research on models of spinal trauma has showed that after transplantation, Schwann cells start to stimulate axonal regeneration and support neuronal survival (6).

There are two types of Schwann cells: myelinating and non-myelinating cells. After establishing axonal contact, both of these cells are capable of dedifferentiation, and they exhibit either myelinating or non-myelinating properties based on axonal signals. For the survival of mature Schwann cells, there is no need for axonal signals, but they are important for the survival of immature and precursor Schwann cells. Mature Schwann cells have a high degree of plasticity, and they are able to regulate their own survival by releasing several growth factors (insulin-like growth factors (IGFs), platelet-derived growth factor-BB (PDGF-BB), and neurotrophin-3 (NT-3)). These growth factors play a crucial role in the autocrine circuit that leads to the inhibition of apoptosis (7). Unfortunately, there is currently no widely recognized, effective treatment for sight-threatening retinal degenerative diseases such as age-related macular degeneration (AMD) and retinitis pigmentosa (RP), which are the leading causes of blindness in the world. Although many studies have attempted to find a treatment for these diseases, there is still no adequate method for promoting photoreceptor survival.

Recently, there has been an interesting proposal to deliver appropriate levels of growth factors to the retinas of patients with retinal degenerative diseases in order to enhance photoreceptor survival. Schwann cells produce several factors necessary for photoreceptor survival, including bFGF (8), GDNF, BDNF (9), and ciliary neurotrophic factor (CNTF) (10). Selection of an appropriate source of cells for transplanting into a patient with retinal degenerative disease has been a major concern because of immune rejection problems and logistic and ethical issues. However, Schwann cells can be obtained from the recipient’s own tissue, and Schwann cell transplants can thereby overcome all of the aforementioned problems. Research has shown that subretinal injection of Schwann cells in dystrophic Royal College of Surgeons (RCS) rats can lead to increases in photoreceptor survival time up to 9 months after the injection. It appears that the release of growth factors (bFGF, GDNF, BDNF, and CNTF (11)) by Schwann cells into the subretinal space promotes photoreceptor survival and the regeneration of damaged neurons. Furthermore, a major strength of using subretinal Schwann cell injections is their persistent functional stability after the injection, which is evidenced by the positive effect they have on visual responsivity in the visual field after the injection.

There are several theories regarding how these Schwann cells survive and support the function of photoreceptors after sub-retinal delivery injection and do not vanish in a new niche. However, the most popular theory is that a sheet rapidly forms between the retinal pigment epithelium and photoreceptors, which allows the Schwann cells to survive and secrete the growth factors. Oxidative stress occurs in the natural course of aging, and it can lead to high levels of intracellular oxidization. When cells are younger, they can overcome oxidative stress by increasing the production of defensive antioxidants (12). However, eventually, the cells stop being able to mitigate the effects of oxidative stress (12). Oxidative stress-induced damage can be accelerated and become more severe as a result of several environmental risk factors including smoking and light exposure. These risk factors have also been associated with increases in the incidence of retinal degenerative diseases such as AMD (13). The damage in AMD is thought to be caused by both reactive oxygen species (ROS) and other agents such as the lipid peroxidation products carboxyethylpyrrole and homocysteine (14, 15). It is believed that antioxidant supplementations and zinc may slow down the progression of retinal degenerative diseases such as AMD (16, 17). Hypoxia, hyperglycemia, and oxidative stress contribute directly and indirectly to Schwann cell dysfunction (18). Schwann cells respond to oxidative stress as a result of the activation of nuclear factor E2-related factor 2 (Nrf2), which is a key transcription factor in the antioxidant response pathway. In addition to the activation of Nrf2, oxidative stress leads to the expression of phase II antioxidant enzymes. Hyperglycemia-induced injury can be attenuated in dorsal root ganglion (DRG) neurons by stimulating caspase 3. Schwann cells can respond to hyperglycemia and oxidative stress by further increasing the expression of antioxidant enzymes (19). This high degree of antioxidant capacity of Schwann cells may help to control ROS generated by fluctuations in blood glucose concentrations (20). Besides Schwann cells, many cells in different parts of body can respond to ROS by initiating an antioxidant response. The endogenous antioxidant enzymes used to detoxify ROS in the nervous system include superoxide dismutase (SOD) (21), heme oxygenase (HO-1) (22), catalase (23), glutathione S-transferase (GST) (24), nicotinamide adenine dinucleotide phosphate-oxidase (NADPH), and quinone oxidoreductase-1 (NQO1) (25).

## Hypotheses

Previous research has highlighted the promising effect of subretinal Schwann cell injections on the survival rate of photoreceptors in the retina (11). The mechanism behind this effect may be the secretion of growth factors in the subretinal space by the Schwann cells (7, 8). However, Schwann cells have proven antioxidant effects in the PNS of patients with diabetes, and Schwann cell-mediated inhibition of oxidative stress in the CNS may underlie the improved retinal function after a subretinal Schwann cell injection. Thus, we hypothesized that the antioxidant capacity of Schwann cells may play a role in reducing degenerative retinal damage. A subretinal injection of Schwann cells may lead to the release of the antioxidant enzymes in response to the oxidative stress present at the injection site as a result of the degenerative retinal damage. Understanding the possible pathways by which Schwann cells reduce CNS damage could help to determine which pathways and enzymes to target in order to reduce CNS damage further.

## Evaluation of the Hypotheses

Schwann cells from the sciatic nerves of neonatal rats will be cultured using a method developed by Brook et al, and the cells will subsequently be purified (26). The retina of each rat will be damaged using an injection of sodium iodide, and then the rat will be anesthetized. Each pupil will be dilated using tropicamide, and a suture will be used to stabilize the eye while the Schwann cells are injected into the dorso-temporal subretinal space by means of either a 30-gauge steel needle or a fine glass capillary attached by tubing to a 10-mL Hamilton syringe. After 8 weeks, a behavioral assessment will be carried out using a head-tracking method based on an optokinetic test invented by Cowey and Franzini (27). The rats will be placed into an enclosed clear plastic container surrounded by a motor-powered drum that can be rotated clockwise and counterclockwise. Perpendicular black-and-white lines will be arranged in three varying widths, subtending 0.125, 0.25, and 0.5 cyc/deg. These three sets of lines will be shown in a random order to the rats, and they will be alternately rotated clockwise and counterclockwise, for 60 s in each direction. This test will stimulate a subcortical reflex, which in turn will stimulate an involuntary head turn in the rats as they track the moving lines. To allow precise assessment of the movements, the tests will be videotaped. For further evaluation, the tests will be repeated at 12 and 20 weeks after the Schwann cell injections. The electrical responses of the retinal cells will be measured using electroretinography. Optical coherence tomography will be used to study the structure of the retinal layers. At 20 weeks after the Schwann cell injections, the rats will be anesthetized and killed, the eyes will be excised, and the activity of antioxidant enzymes (i.e., SOD, HO-1, catalase, and GST) will be measured using enzyme-linked immunosorbent assays. 

## DISCUSSION

**Figure 1:**
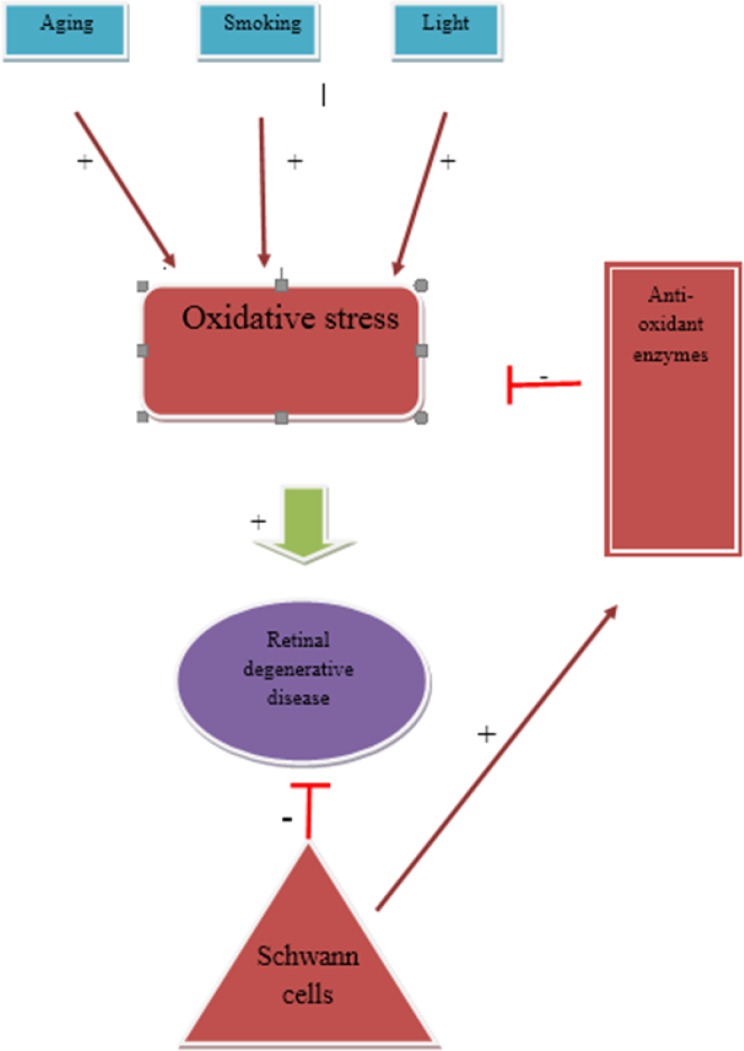
Previously identified pathways linked by novel connections that may explain the underlying mechanism of Schwann cell-mediated preservation of vision in retinal degenerative diseases.

Schwann cells have been shown to exhibit remyelination capabilities in both the PNS and CNS (2). These cells have also been shown to secrete growth factors that increase the survival rate of retinal cells (5). Degenerative retinal diseases are a major concern in many developed countries as a result of their aging populations. One of the most widely known pathways responsible for degenerative retinal diseases is the oxidative stress pathway (13). Increasing the levels of antioxidant enzymes and other factors at the site of the damage could reduce the rate of retinal degeneration. In patients with diabetes, Schwann cells have been shown to protect the PNS from oxidative stress that can result from the ingestion of large amounts of sugar. Schwann cells can also prevent the development of diabetic neuropathy in the PNS by activating antioxidant enzymes (20). Figure 1 helps to explain our hypothesis by showing an innovative diagram of the previously identified pathways linked by novel connections that may explain the underlying mechanism of Schwann cell-mediated preservation of vision in retinal degenerative diseases. In addition to the previously identified pathways, like growth factor secretion, the ability of Schwann cells to protect against oxidative stress in the PNS may also reflect the mechanism by which retinal cells can be protected by injected Schwann cells in patients with degenerative diseases. By increasing our understanding of the exact mechanisms involved in the protective role of Schwann cells in the CNS, this protective ability could eventually be augmented by introducing antioxidant enzymes to the damaged sites.

## Overview Box

**First question:** What do we already know about the subject?

Schwann cells can protect the central nervous system and remyelinate injured neurons by secreting growth factors. In addition, Schwann cells can reduce the adverse effects of oxidative stress in the peripheral nervous system by increasing the expression of antioxidant enzymes. **Second question:** What does your proposed theory add to the current knowledge available, and what benefits does it have?

Understanding the possible mechanism by which Schwann cells can improve the function of injured cells in the CNS may result in better management of retinal degenerative diseases. For example, if the inhibition of oxidative stress is one of the major pathways activated after subretinal Schwann cell injections, the use of these cells in combination with antioxidants for the treatment of early-stage degenerative retinal diseases may inhibit the progression of these diseases.

**Third question:** Among numerous available studies, what special further study is proposed for testing the idea?

The most important issue is to distinguish the exact antioxidants that play a role in the preservation of vision by Schwann cells after subretinal injections. Further studies could reveal which cells in the retina respond better to the antioxidant effects, and these studies may shed light on how to develop new treatments for currently incurable degenerative retinal diseases.
